# Fatal Feline Leukemia Virus-Associated Enteritis in a Wild Eurasian Lynx (*Lynx lynx*) in Germany †

**DOI:** 10.3390/biology13120997

**Published:** 2024-11-30

**Authors:** Katharina M. Gregor, Monica Mirolo, Florian Brandes, Sonja T. Jesse, Franziska Kaiser, Jutta Verspohl, Sybille Wölfl, Albert D. M. E. Osterhaus, Wolfgang Baumgärtner, Martin Ludlow, Andreas Beineke

**Affiliations:** 1 Department of Pathology, University of Veterinary Medicine Hannover, 30559 Hannover, Germany; katharina.manuela.gregor@tiho-hannover.de (K.M.G.); wolfgang.baumgaertner@tiho-hannover.de (W.B.); 2 Research Center for Emerging Infections and Zoonoses, University of Veterinary Medicine Hannover, 30559 Hannover, Germany; monica.mirolo@tiho-hannover.de (M.M.); sonja.jesse@twincore.de (S.T.J.); franziska.kaiser@nih.gov (F.K.); albert.osterhaus@tiho-hannover.de (A.D.M.E.O.); martin.ludlow@tiho-hannover.de (M.L.); 3 Wildtier- und Artenschutzstation e.V., 31553 Sachsenhagen, Germany; florian.brandes@wildtierstation.de; 4 Institute of Microbiology, University of Veterinary Medicine Hannover, 30173 Hannover, Germany; jutta.verspohl@tiho-hannover.de; 5 Luchs Bayern e.V., 93449 Waldmünchen, Germany; sybille.woelfl@luchs-bayern.de

**Keywords:** feline leukemia virus, Eurasian lynx, wildlife, FeLV-associated enteritis, secondary bacterial infection

## Abstract

The Eurasian lynx (*Lynx lynx*), a widespread wild felid on the Eurasian continent, is currently classified as “critically endangered” in Germany. Understanding the impact of infectious agents is of particular importance for the continued conservation of these animals, especially regarding pathogens with broad host ranges and risk of interspecies transmission. Feline leukemia virus (FeLV) is known to infect wild and domestic felids worldwide, including several species of lynx, but it has not been reported thus far in the Eurasian lynx. In September 2020, a 16-month-old female Eurasian lynx from the Bavarian Forest, Germany, showed a sudden onset of gastrointestinal signs such as anorexia, diarrhea, and vomiting, and died within one week. Macroscopic and histologic examination revealed hemorrhagic-necrotizing enteritis and typhlocolitis, with the degeneration of crypts and crypt abscesses, as well as depleted Peyer’s patches. In addition, the animal showed lymphoid depletion (lymph nodes, thymus, and spleen) and hypocellularity of the bone marrow. FeLV infection was confirmed by immunohistochemistry and next generation sequencing. A secondary bacterial infection with hemolytic *Escherichia coli* and *Clostridium perfringens* type A was present in the intestine. This is the first report of FeLV-associated enteritis, lymphoid depletion and bone marrow suppression with associated secondary bacterial infection in a Eurasian lynx.

## Simple Summary

The Eurasian lynx, critically endangered in Germany, faces new threats from diseases like feline leukemia virus (FeLV). In September 2020, a young 16-month-old female lynx from the Bavarian Forest in Germany developed sudden anorexia, diarrhea, and vomiting, and died within a week. An examination revealed severe intestinal lesions and a weakened immune system. Tests confirmed that the lynx was infected with FeLV, which can cause immunosuppression, making animals vulnerable to other infections. In this case, harmful bacteria, including *Escherischia coli* and *Clostridium perfringens*, were also found in its intestines. This marks the first reported case of FeLV-associated enteritis in a Eurasian lynx, highlighting a new potential risk for this animal species. Understanding how FeLV affects the Eurasian lynx is important for future conservation efforts.

## 1. Introduction

Feline leukemia virus (FeLV) is an immunosuppressive and oncogenic, diploid single-stranded, positive-sense RNA virus belonging to the genus *Gammaretrovirus* of the family *Retroviridae* that affects wild and domestic felids worldwide [1]. Depending on the susceptibility of the host, virus strain, virus subgroup (FeLV A, B, C, D, T) and infectious dose, FeLV-induced disease can have different manifestations, with progressive, regressive, abortive, or atypical disease courses [1,2]. Several disorders have been associated with persistent FeLV infection, including immunosuppression and bone marrow suppression, neoplasia, neuro- and enteropathies, reproductive failure and immune-mediated disorders, as well as fading kitten syndrome [1,3]. Virus transmission occurs mainly horizontally via saliva and other body fluids, as well as fleas or iatrogenic means. Vertical transmission is frequently seen in persistently infected cats or, less commonly, in regressively or focally atypically infected cats, with feral domestic cats (*Felis catus*) playing a prominent role in the spread of FeLV among felids [1,4,5,6,7]. In addition to domestic cats and wild cats (*Felis silvestris silvestris*), the Eurasian lynx (*Lynx lynx*) represents another potential host in Germany [8].

The Eurasian lynx is a widespread wild felid on the Eurasian continent, whose populations in Central and Western Europe are highly fragmented with approximately 8000 to 9000 individuals (excluding Russia and Belarus) [9]. By 1900, the Eurasian lynx had been extinct throughout Western and Southern Europe due to direct persecution by humans. Its return to Central Europe can be attributed to governmental repopulation efforts. However, the lynx population in Germany is still small and isolated and currently classified as “critically endangered”, with subpopulations present in the Bavarian forest (belonging to the Bohemian–Bavarian–Austrian population), the Harz Mountains and Palatinian forest [10,11,12,13,14,15]. Apart from anthropogenic factors (illegal killing, fatal road accidents), their population viability is inter alia affected by the small numbers of individuals and diseases [14]. The Eurasian lynx has been shown to be susceptible to infections with several pathogens, potentially as a result of contact with other animals [16,17,18,19,20]. Continued investigations into the etiology of diseases and causes of death are therefore important for the long-term conservation efforts for this species.

Free-ranging and captive wild felid populations, including cheetahs (*Acinonyx jubatus*) [21,22,23], Pallas’ cats (*Felis [Otocolobus] manul*) [24], sand cats (*Felis margarita*) [25], guignas (*Leopardus guigna*) [5], ocelots (*Leopardus pardalis*) [26], oncillas (*Leopardus tigrinus*) [26], bobcats (*Lynx rufus*) [27], Amur leopards (*Panthera pardus orientalis*) [28], jaguars (*Panthera onca*) [29], leopard cats (*Prionailurus bengalensis*) [28,30,31], North American pumas (*Puma concolor*) [4,7], and jaguarundis (*Puma yagouaroundi*) [32], have been tested positive for an FeLV infection but rarely diagnosed with an FeLV-associated disease [22,27,32]. The European wild cat [33,34,35,36,37,38] and Iberian lynx (*Lynx pardinus*) [6] are the most affected species in Europe. In Germany, the most recent prevalence of FeLV infection in domestic cats was 1.8% for virus antigens and 16.4% for virus-specific antibodies [39]. In contrast, the prevalence of FeLV infection in wild cats appears to be significantly higher, as in a 1999 study in Germany in which 49% of animals were positive for virus antigens and 75% for antibodies [37]. Similar values were also observed in a more recent study in Luxembourg, with a prevalence of 52.9% for antigens [36]. No evidence of FeLV infections have been reported so far for the Eurasian lynx in Germany [8]. This present report describes the first case of fatal FeLV-associated enteritis in a Eurasian lynx from Germany and phylogenetic characterization of the virus.

## 2. Case Report

A four-month-old orphaned female Eurasian lynx was captured on a farmstead in the Bavarian Forest, Germany, in September 2019 and brought to the wildlife rescue and conservation center, Sachsenhagen, for rehabilitation in October 2019. During the quarantine, it was tested positive for FeLV in November 2019, with both p27 antigen by ELISA (NovaTec VetLine FeLV Antigen ELISA, NovaTec Immundiagnostica GmbH, Dietzenbach, Germany; performed by LABOKLIN) and FeLV-RNA by RT-PCR (performed by LABOKLIN) detected in the serum [40]. Thereafter, the lynx was kept in a single enclosure in Sachsenhagen.

Hematology and serum chemistry performed in August 2020 revealed only minor deviations (Table S1), while p27 antigen was repeatedly detected in the serum by ELISA (performed by LABOKLIN) as well as FeLV-RNA by RT-PCR (performed by LABOKLIN). In addition, FeLV RNA was detected in saliva (Ct value 21.2) and feces (Ct value 22.3) by RT-PCR (reference: ≥45; performed by Clinical Laboratory, Vetsuisse Faculty, University of Zurich) [41]. ELISA yielded a serum antibody titer of 4.68 (reference: <9) for the feline coronavirus (FCoV; NovaTec VetLine FCoV Antibody ELISA, NovaTec Immundiagnostica GmbH, Dietzenbach, Germany; performed by LABOKLIN). No serum antibodies against the feline immunodeficiency virus (FIV) were detected by ELISA (NovaTec VetLine FIV Antibody ELISA, NovaTec Immundiagnostica GmbH, Dietzenbach, Germany; performed by LABOKLIN). In September 2020, the lynx exhibited a sudden onset of anorexia and diarrhea. After one week, the animal developed foamy emesis and was found dead in the enclosure.

**Figure 1:**
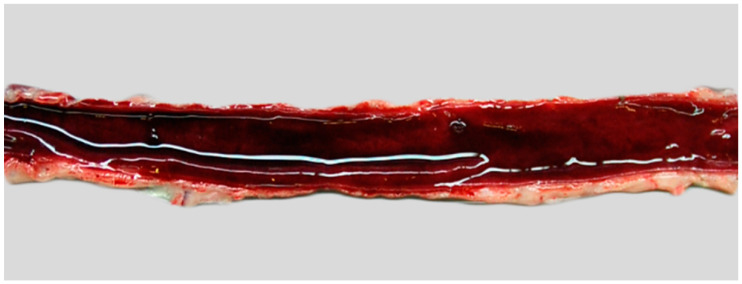
Macroscopic findings in a Eurasian lynx (*Lynx lynx*). The jejunum is diffusely red and filled with blood-tinged digesta.

At necropsy, the lynx was in a good nutritional state. The stomach was empty, and the jejunum and ileum showed a red mucosa and contained small amounts of red, cloudy, liquid ingesta (Figure 1). Occasionally, depleted Peyer’s patches were observed. The caecum and colon exhibited diffusely red mucosa and small amounts of coagulated blood. Dilatation of the colon was found, and the rectum was moderately filled with coagulated blood. Mesenteric lymph nodes were enlarged and diffusely dark red. The subcutis was dry and showed multiple hemorrhages. In the lung, multifocal, raised, firm foci with a diameter of up to 0.5 cm as well as dark red, non-raised areas with a diameter of about 0.4 cm were present. The bone marrow was diffusely red.

**Figure 2:**
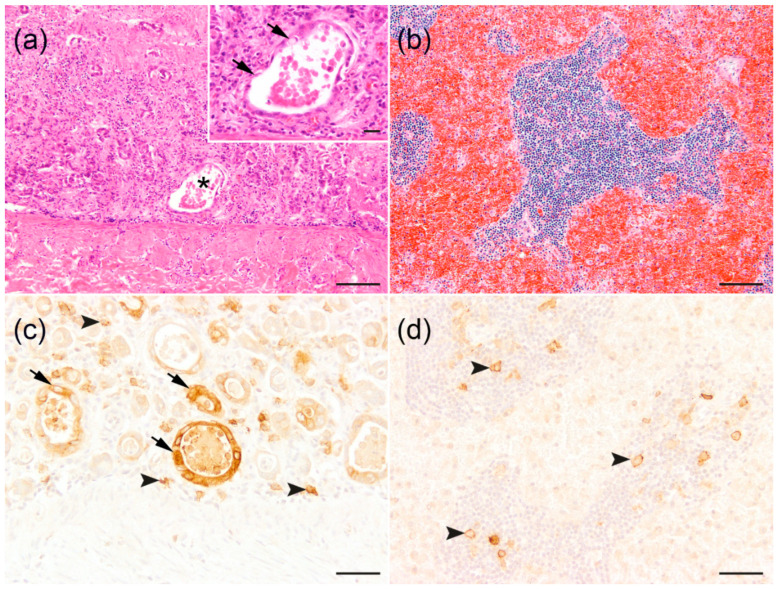
Histologic and immunohistochemical findings in small intestine and mesenteric lymph node of a Eurasian lynx (*Lynx lynx*). (**a**) Severe, diffuse, subacute, hemorrhagic-necrotizing enteritis with loss of intestinal crypts, crypt dilatation (star), and crypt epithelial degeneration. Hematoxylin–eosin (HE). Bar = 100 µm. Insert. Dilatation of a single intestinal crypt with crypt epithelial degeneration (arrows) and luminal accumulation of cellular debris (crypt abscess). HE. Bar = 20 µm. (**b**) Lymphatic depletion and blood resorption in mesenteric lymph node. HE. Bar = 100 µm. (**c**) Immunolabeling of feline leukemia virus gp85/gp70 envelope protein in the intestinal crypt epithelium (arrows) and infiltrating mononuclear cells (arrowheads). Bars = 50 µm. (**d**) Immunolabeling of feline leukemia virus gp85/gp70 envelope protein in mononuclear cells (arrowheads) in mesenteric lymph node. Bar = 50 µm.

Histological examination was performed on the formalin-fixed and paraffin-embedded specimens of respective organs, stained with hematoxylin and eosin (HE). A severe, diffuse, hemorrhagic-necrotizing enteritis and typhlocolitis, with the dilatation and degeneration of crypts, multiple crypt abscesses, and depleted germinal centers in Peyer’s patches was observed (Figure 2a). The thymus was markedly atrophied. Mandibular, lung, and mesenteric lymph nodes as well as spleen exhibited lymphatic depletion. In addition, moderate blood resorption was observed in the mesenteric lymph nodes (Figure 2b). The sternal and femoral bone marrows were hypocellular. Multifocal granulomatous pneumonia with intralesional nematode larvae was detected in the lung. The adrenal glands showed mild, multifocal, cortical hemorrhages. Furthermore, an increased urea content of 250 mg/dL (reference: <50 mg/dL) was identified in the aqueous humor of the eye.

Immunohistochemistry of the small and large intestines and mesenteric lymph nodes was performed for the detection of FeLV (anti-gp85/70 envelope protein, clone C11D8, Custom Monoclonals International Corp., Sacramento, CA, USA, mouse, monoclonal, 1:200), feline panleukopenia virus (FPV)/canine parvovirus (CPV; anti-capsid immunodominant region #1, clone CPV1-2A1, Custom Monoclonals International Corp., Sacramento, CA, USA, mouse, monoclonal, 1:500), and feline coronavirus (FCoV; anti-coronavirus nucleoprotein; clone FIPV3-70, Custom Monoclonals International Corp., Sacramento, CA, USA, mouse, monoclonal, 1:10,000) using the avidin–biotin peroxidase complex (#PK 6100, Vectastain elite ABC kit, Vector Laboratories, Burlingame, CA, USA). 3,3′-diaminobenzidine tetrahydrochloride was used as a chromogen and Mayer’s haematoxylin as a counterstain, as previously described [42]. As negative controls, primary antibodies were substituted with ascites fluid from non-immunized BALB/c mice (1:1000; Cedarlane, Burlington, NC, USA). An FeLV envelope protein was detected intralesionally within the intestinal crypt epithelium, as well as in the mononuclear cells of the intestinal lamina propria and mesenteric lymph nodes (Figure 2c,d and Figure S1). No antigen for FPV/CPV or FCoV was detected.

Bacterial culture revealed a high bacterial count of hemolytic *Escherichia coli* and a medium bacterial count of *Clostridium perfringens* type A (without ß2 toxins) in the colon.

**Figure 3:**
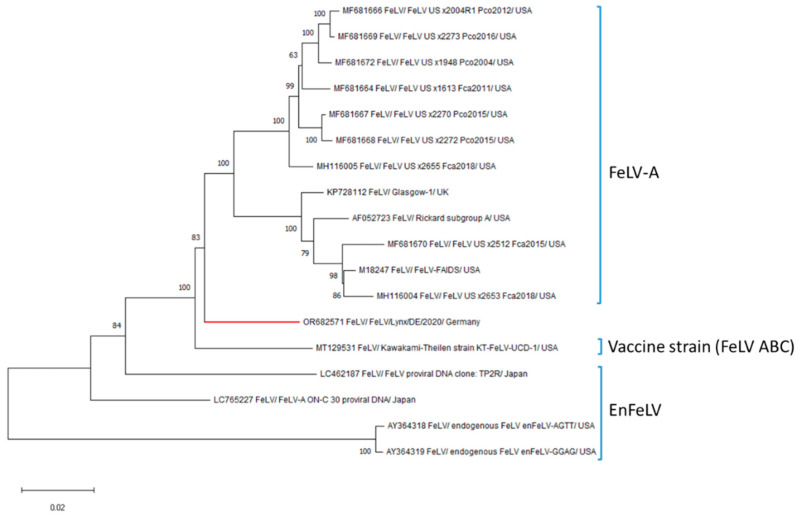
Phylogenetic analysis based on the complete coding sequence of feline leukemia virus (FeLV). The tree was constructed using the maximum likelihood method and the GTR + G was used as best fit model to shape evolutionary rates across sites with 1000 bootstraps. Bootstrap values are presented at nodes. The scale bar indicates the number of nucleotide changes per site. Taxon names are presented by the GenBank accession number, virus name and strain. Genome sequences belonging to different FeLV clades were downloaded from GenBank (Table S4).

Pooled intestine and lymph node samples were used for metagenomics analysis via next generation sequencing (NGS). Details on tissue processing, NGS, as well as the phylogenetic and recombination analyses can be found in the Supplementary Material (S1. Tissue processing and next generation sequencing; S2. Phylogenetic and recombination analyses). Analysis of the resulting data using the CZ-ID metagenomics pipeline showed the presence of 133 reads with homology to FeLV, subgroup A (GenBank accession number MF681672). The read reference assembly using CLC Genomics Workbench v12.0 resulted in a 93.1% coverage of the FeLV genome (Figure S2a). Genome gaps in the partial FeLV genome sequence of 8360 bp was completed by Sanger sequencing using primers designed on the available sequence retrieved by NGS (Tables S2 and S3; Figure S2b–d). The full-length genome was deposited in Gen bank with the accession number OR682571. Maximum likelihood phylogenetic analyses using neighbor joining method based on whole FeLV genome coding sequences and individual envelope coding sequences of the German FeLV strain were performed to determine its evolutionary relationship to other FeLV strains. Analysis of the phylogenetic tree generated based on complete coding sequences showed that the FeLV strain FeLV/Lynx/DE/2020 detected in the German lynx clusters with other members of the FeLV-A subgroup (Figure 3). Moreover, analysis of the phylogenetic tree based on the envelope genes shows that the FeLV strain from this study clusters with FeLV-A viruses (Figure S3). The FeLV FeLV/Lynx/DE/2020 strain shares 95.03% nucleotide sequence homology to a FeLV detected in a domestic cat in Florida, USA (FeLV_US_x2270_Pco2015, GenBank accession no. MF681667) [4]. Recombination analyses did not identify recombination events in the German FeLV strain [43].

## 3. Discussion

The cause of the sudden death of the lynx was determined to be hemorrhagic-necrotizing enteritis and typhlocolitis due to progressive FeLV infection with secondary bacterial infection. Aside from FeLV, FPV/CPV, and FCoV, viruses that can cause diarrhea in cats include adenovirus, astrovirus, feline calicivirus, rotavirus, torovirus, FIV, feline kobuvirus, and feline norovirus [44,45,46,47]. The most likely differential diagnoses in the present case, FPV/CPV and FCoV infections, were ruled out by immunohistology. In addition, NGS did not reveal any evidence of viruses other than FeLV.

Similar to FeLV-associated enteritis (FAE) in domestic cats, the investigated lynx showed clinical signs such as anorexia, vomiting, and dehydration. FeLV-induced intestinal lesions with intestinal crypt alterations also represent the typical findings in domestic cats following an FeLV infection [46,47,48]. Similar lesions were reported in an FeLV-infected jaguarundi, which showed weight loss, vomiting, anorexia, and diarrhea [32]. The investigated lynx was younger than the domestic cats affected by FAE (usually >2 years). Furthermore, domestic cats with FAE show a normocellular or hypercellular bone marrow rather than a hypocellular bone marrow as found in the present case [46,47,48].

Lymphoid depletion observed in the thymus, lymph nodes, spleen, and Peyer’s patches indicates concomitant immunosuppression, while hypocellularity of the hematopoietic cells suggests bone marrow suppression [49]. Both immunosuppression (bobcat, North American puma, jaguarundi) [4,27,32] and bone marrow suppression (Iberian lynx, bobcat) [6,27,50] have been reported in FeLV-infected wild felids.

In the present case, hemolytic *Escherichia coli* and *Clostridium perfringens* type A were present in the intestine. Both bacteria can be found as commensals in the intestine of healthy cats [51,52], which likely caused a progression of enteritis with fatal consequences following the FeLV-induced immunosuppression and intestinal alterations.

Investigation of the evolutionary origin of FeLV strains has been particularly challenging due to the lack of published sequences of endogenous and exogenous FeLV strains originating in Europe, leading to consequent sampling bias. For this reason, additional bioinformatic analyses of the recovered sequences were carried out to minimize errors. Analysis of a phylogenetic tree based on the available full-length FeLV genomes showed that the German FeLV strain forms a unique separate clade from multiple FeLV viruses from subgroup A. However, a phylogenetic tree based on the individual FeLV envelope genes showed that the German FeLV clusters with the FeLV of subgroup A, suggesting that this virus belongs to the FeLV-A subgroup of the non-recombinant FeLV strains. This was supported by recombination analysis using the available FeLV sequences. Recombination analysis failed to identify potential parental donor strains. The origin of the infection in the lynx cannot be conclusively clarified. Contact with infected feral domestic cats or wild cats could have been the source of the FeLV infection, in addition to other lynx [4,7,50,53]. In this case, however, the most probable cause is the contact with FeLV-infected domestic cats, as wild cats do not occur in this region of Bavaria, and FeLV is currently not considered endemic in the German lynx population.

Caution is required for the German Eurasian lynx in view of its geographical confinement, the relatively low genetic diversity in individual populations [54,55], as well as the possible emergence of more aggressive FeLV strains. This becomes important also in light of anecdotal evidence that, apart from this case, two other lynx were found to be infected with FeLV—one in Switzerland (2019, Jura population) and another in Germany (2020, Harz Mountain population) [56,57]. Previous reports of mange in the Eurasian lynx in the Harz Mountains [57] and Switzerland [20], canine morbillivirus (formerly canine distemper virus) in the Harz Mountains [17,56], and FeLV in the Iberian lynx [58] show that infectious diseases can suddenly become critical. Thus, additional monitoring and veterinary screening of European lynx populations is warranted, along with the further molecular characterization of circulating FeLV strains.

## 4. Conclusions

In conclusion, this is the first reported case of FeLV-associated disease in a Eurasian lynx. Given that the Eurasian lynx is considered “critically endangered” in Germany, knowledge about potential infectious agents is vital. As there is no information on the occurrence of FeLV in Eurasian lynx, its epidemiological role is currently unclear. Future studies are warranted to investigate the prevalence and tissue tropism of FeLV, as well as its circulating genotypes in wild life.

## Data Availability

The original contributions presented in the study are included in the article/Supplementary Material, further inquiries can be directed to the corresponding author.

## Supplementary Materials

The following supporting information can be downloaded at: https://www.mdpi.com/article/10.3390/biology13120997/s1, S1: Tissue processing and next generation sequencing; S2: Phylogenetic and recombination analyses; Table S1: Hematology and serum chemistry of a Eurasian lynx (*Lynx lynx*) one month prior to its death; Table S2: PCR primers; Table S3: Sanger sequencing primers; Table S4: Sequences used for phylogenetic and recombination analyses; Figure S1: Immunohistochemical findings of negative controls in small intestine and mesenteric lymph node of Eurasian lynx (*Lynx lynx*); Figure S2: Detection of gap filling of FeLV genome; Figure S3: Phylogenetic analyses of FeLV envelope genes. References [43,59,60,61,62,63,64,65,66] are cited in the supplementary materials.
