# Supplementary material for: Fatal Feline Leukemia Virus-Associated Enteritis in a Wild Eurasian Lynx (Lynx lynx) in Germany
Source: Biology (Basel). 2024 Nov 30;13(12):997. doi: 10.3390/biology13120997 (PMC11727347; doi:10.3390/biology13120997)
Supplement: Supplementary file 1 [file biology-13-00997-s001.zip › biology-3306167-supplementary.pdf]

biology

MDPI

Case Report

Fatal feline leukemia virus-associated enteritis in a wild Eurasian lynx (*Lynx lynx*) in Germany

Katharina M. Gregor[1,#], Monica Mirolo[2,#], Florian Brandes[3], Sonja T. Jesse[2], Franziska Kaiser[2], Jutta Verspohl[4], Sybille Wölfl[5], Albert D.M.E. Osterhaus[2], Wolfgang Baumgärtner[1], Martin Ludlow[2,§], Andreas Beineke[1,§,*]

[1] Department of Pathology, University of Veterinary Medicine Hannover, Germany; katharina.manuela.gregor@tiho-hannover.de (K.M.G.); Wolfgang.Baumgaertner@tiho-hannover.de (W.B.)
[2] Research Center for Emerging Infections and Zoonoses, University of Veterinary Medicine Hannover, Germany; Monica.Mirolo@tiho-hannover.de (M.M.); sonja.jesse@twincore.de (S.T.J.); franziska.kaiser@nih.gov (F.K.); Albert.Osterhaus@tiho-hannover.de (A.D.M.E.O.); Martin.Ludlow@tiho-hannover.de (M.L.)
[3] Wildtier- und Artenschutzstation e.V., Sachsenhagen, Germany; florian.brandes@wildtierstation.de (F.B.)
[4] Institute of Microbiology, University of Veterinary Medicine Hannover, Germany; jutta.verspohl@tiho-hannover.de (J.V.)
[5] Luchs Bayern e.V., Waldmünchen, Germany; sybille.woelfl@luchs-bayern.de (S.W.)
* Correspondence: andreas.beineke@tiho-hannover.de
# Equal first authors
§ Equal senior authors

Supplementary material

S1. Tissue processing and next generation sequencing

S2. Phylogenetic and recombination analyses

Table S1. Hematology and serum chemistry of Eurasian lynx (*Lynx lynx*) one month prior to its death

Table S2. PCR primers

Table S3. Sanger sequencing primers

Table S4. Sequences used for phylogenetic and recombination analyses

Figure S1. Immunohistochemical findings of negative controls in small intestine and mesenteric lymph node of Eurasian lynx (*Lynx lynx*)

Figure S2. Detection of gap filling of FeLV genome

Figure S3. Phylogenetic analyses of FeLV envelope genes

S1. Tissue processing and next generation sequencing

A pool of frozen intestinal and lymph node tissue from the lynx under investigation (approximately 50 mg) was homogenized in 500 µl PBS and centrifuged for 5 minutes at 12,000 relative centrifugal force (RCF). The resulting supernatant was sieved using a 0.45 µm spin filter (Merck) for 5 minutes at 12,000 RCF. RNA was extracted using the TRIzol Reagent (Thermo Fisher Scientific) as per manufacturer instructions and then stored at -80°C. Conversion to cDNA was performed using Superscript IV (Thermo Fischer Scientific) with non-ribosomal hexamers as per protocol guidelines. Double-stranded DNA (dsDNA) was generated from cDNA by Klenow fragment (NEB, Ipswich, MA, USA), followed by random PCR amplification using a sequence-independent, single-primer amplification (SISPA) protocol [1]. PCR products were purified using a Monarch PCR/DNA Cleanup kit (NEB), and the DNA library was prepared using a Nextera XT DNA Library Preparation Kit (Illumina, Inc, San Diego, CA, USA), followed by sequencing on an Illumina NextSeq 550 system to obtain 2 × 150 bp reads, with a (paired end) per sample. Raw sequencing data (FASTQ files) were initially analyzed using the CZ-ID bioinformatics pipeline to identify the sequencing reads with homology to known viruses [2]. A consensus genome was then generated using CLC Genomics Workbench v12.0 (Qiagen GmbH, Hilden, Germany) by read mapping to the genome sequence of Feline leukemia virus, subgroup A (FeLV-FAIDS) (GenBank accession no. MF681672). The genome sequence was completed by Sanger sequencing of the RT-PCR amplicons generated using primers based on the initial consensus sequence obtained by next-generation sequencing (NGS) (Table S2 and S3). All RT-PCR reactions were performed using Q5® High-Fidelity PCR Kit (NEB). Genome annotations were performed using Geneious Primer software (Biomatters, Ltd., Auckland, New Zealand). Subsequently, RNA which had been extracted from frozen brain tissue and a pool of spleen and liver tissue was reverse transcribed using Superscript IV (Thermo Fischer Scientific). The resulting cDNA was used for PCRs performed with a Q5® High-Fidelity PCR Kit (NEB) and with previously published FeLV primers [3] at an annealing temperature of 48°C. The reaction mixture without the presence of any nucleic acid served as a negative control.

S2. Phylogenetic and recombination analyses

Genome sequences belonging to different FeLV clades were downloaded from GenBank (Table S4). The evolutionary relationship between the FeLV strains was investigated using the whole coding sequence and envelope genes. A multiple sequence alignment of FeLV strains was first generated using MAFFT version 7 [4]. Maximum likelihood phylogenetic trees of the complete polyprotein and individual envelope genes and best fit models for all alignments were calculated using MEGA11 with 1000 bootstrap values [5]. The phylogenetic relationship of FeLV envelope gene sequences was analyzed separately, due to the potential for recombination events between these genes of exogenous FeLV-A and endogenous FeLV during viral reverse transcription and integration [6]. Recombination analyses were performed with RDP4 software [7], which included the algorithms RDP, GeneConv, Bootscan, MaxChi, Chimera, SiScan, and 3Seq, with an initial cut-off p-value of 0.01. Events recognized by at least three of the algorithms and with $p < 0.05$ were selected for further confirmation analyses of recombination.

Table S1. Hematology and serum chemistry of a Eurasian lynx (*Lynx lynx*) one month prior to its death.

Parameter (unit)	Value	Reference [8]
	**Hematology**	
**Erythrocytes (x10^6/µl)**	**10.7**	**6.77-10.40**
Hematocrit (%)	43	32-50 [9]
Hemoglobin (g/dl)	16.1	10.2-16.5
White blood cells (x10^3/µl)	4.3	1.5-11.4
Lymphocytes (x10^3/µl)	1.9	0.59-2.22
Monocytes (x10^3/µl)	0.0	0.1-0.7
Neutrophils (x10^3/µl)	1.1	0.60-8.13
Eosinophiles (x10^3/µl)	1.2	0.09-2.24
Platelets (x10^3/µl)	401	251-729
	**Serum chemistry**	
Total protein (g/dl)	6.97	5.9-8.4
**ALP (U/l)**	**98**	**10–84**
**AST (U/l)**	**14.2**	**15–160**
ALT (U/l)	18.8	18–128
**CK (U/l)**	**149**	**254–655**
Urea (mg/dl)	61.26	46.0-147.9
Creatinine (mg/dl)	2.036	1.3-2.98
Cholesterol (mg/dl)	108.262	73–163
Triglyceride (mg/dl)	54.87	11–87
Sodium (mmol/l)	152	147–164
Potassium (mmol/l)	4.3	3.6–4.9
Calcium (mmol/l)	2.5	2.13–2.62
Iron (µmol/l)	24.9	9.0–22.6
Albumin (g/l)	45.8	43.6–51.0
Globulin (g/l)	23.9	20.7-39.2
Albumin/globulin ratio	1.9	1.0-2.3

Deviated parameters are indicated in bold. High values are indicated in red. Low values are indicated in blue.

Table S2. PCR primers.

Primers						Product (bp)	Ta[1] (°C)
**Forward primer name**	**gene**	**Sequence (5´-3´)**	**Reverse primer name**	**gene**	**Sequence (5´-3´)**		
FeLV_1f 407-426	5´ untranslated region	GCATCTGACTCGTGGTCTCG	FeLV 6r_2665-2685	gag-pol polyprotein gene	CCTTACCGGTTGCCAGTTGC	2273	66
FeLV 7f 4157-4176	gag-pol polyprotein gene	CTCATTACCAAGCTATGCTC	FeLV 10rvs 5804-5824	gag-pol polyprotein gene	CGAGGTTCTTGGTTTGATGTC	1623	62
FeLV 9 frw 4892-4912	gag-pol polyprotein gene	GAGGTACTTGGGAGTACCAAG	FeLV 10rvs 5804-5824	gag-pol polyprotein gene	CGAGGTTCTTGGTTTGATGTC	943	65
FeLV 8 Frw 5704-5725	gag-pol polyprotein gene	GAAGAGATCCAGAGAGCTCTAG	FeLV 11_7006-7026 rvs	envelope gene	GTTGCGGAGTAGATAGGCAGG	1331	65

[1]annealing temperature.

Table S3. Sanger sequencing primers.

Sequencing primers		Gene
**Name**	**Sequence (5´-3´)**	
FeLV_1f 407-426	GCATCTGACTCGTGGTCTCG	5´ untranslated region
FeLV 2 rvs_741 -760	CGGATAGGGACATTCAGGTAC	5´ untranslated region
FeLV 3f_1144-1146	GTTCCTTACATTACCACATG	gag-pol polyprotein gene
FeLV 4r_1752-1771	CATTGGGTAGTTGGGTTGGC	gag-pol polyprotein gene
FeLV 5f_2224-2242	GAGATGACTAAAGTTCTGG	gag-pol polyprotein gene
FeLV 6r_2665-2685	CCTTACCGGTTGCCAGTTGC	gag-pol polyprotein gene
FeLV 7f 4157-4176	CTCATTACCAAGCTATGCTC	gag-pol polyprotein gene
FeLV 9 frw 4892-4912	GAGGTACTTGGGAGTACCAAG	gag-pol polyprotein gene
FeLV 10rvs 5804-5824	CGAGGTTCTTGGTTTGATGTC	gag-pol polyprotein gene
FeLV 9 frw 4892-4912	GAGGTACTTGGGAGTACCAAG	gag-pol polyprotein gene
FeLV 10rvs 5804-5824	CGAGGTTCTTGGTTTGATGTC	gag-pol polyprotein gene
FeLV 8 Frw 5704-5725	GAAGAGATCCAGAGAGCTCTAG	gag-pol polyprotein gene
FeLV 11_7006-7026 rvs	GTTGCGGAGTAGATAGGCAGG	gag-pol polyprotein gene

Numbers of the primers refer to the consensus genome.

Table S4. Sequences used for phylogenetic analyses and recombination analyses.

GenBank accession no.	Virus strain	Subgroup	Country of origin
MT129531	Feline leukemia virus Kawakami-Theilen strain KT-FeLV-UCD-1	ABC	USA
AF052723	Feline leukemia virus strain Rickard subgroup A	A	USA
KP728112	Feline leukemia virus strain Glasgow-1	A	UK
M18247	Feline leukemia virus, subgroup A (FeLV-FAIDS)	A	USA
MF681664	Feline leukemia virus isolate FeLV_US_x1613_Fca2011	A	USA
MF681665	Feline leukemia virus isolate FeLV_US_x2004_Pco2010	A	USA
MF681666	Feline leukemia virus isolate FeLV_US_x2004R1_Pco2012	A	USA
MF681667	Feline leukemia virus isolate FeLV_US_x2270_Pco2015	A	USA
MF681668	Feline leukemia virus isolate FeLV_US_x2272_Pco2015	A	USA
MF681669	Feline leukemia virus isolate FeLV_US_x2273_Pco2016	A	USA
MF681670	Feline leukemia virus isolate FeLV_US_x2512_Fca2015	A	USA
MF681672	Feline leukemia virus isolate FeLV_US_x1948_Pco2004	A	USA
MH116004	Feline leukemia virus isolate FeLV_US_x2653_Fca2018	A	USA
MH116005	Feline leukemia virus isolate FeLV_US_x2655_Fca2018	A	USA
AY364318	Endogenous feline leukemia virus enFeLV-AGTT	EnFeLV	USA
AY364319	Endogenous feline leukemia virus enFeLV-GGAG	EnFeLV	USA
LC462187	Feline leukemia virus proviral DNA, clone: TP2R	EnFeLV	Japan
LC765227	Feline leukemia virus FeLV-A_ON-C_30 proviral DNA	EnFeLV	Japan
MW839565	Feline leukemia virus isolate CHINA/HLJ/HB	EnFeLV	China

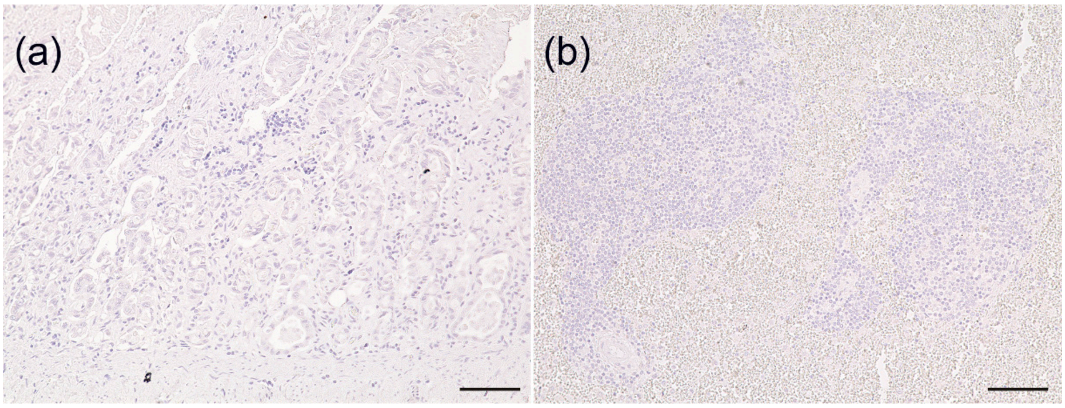

Figure S1. Immunohistochemical findings of negative controls in small intestine and mesenteric lymph node of a Eurasian lynx (*Lynx lynx*). a) No immunolabeling detected with ascites fluid from non-immunized BALB/c mice in the intestinal crypt epithelium or infiltrating mononuclear cells. Bar = 50µm. b) No immunolabeling detected with ascites fluid from non-immunized BALB/c mice in mononuclear cells in mesenteric lymph node. Bar = 50µm.

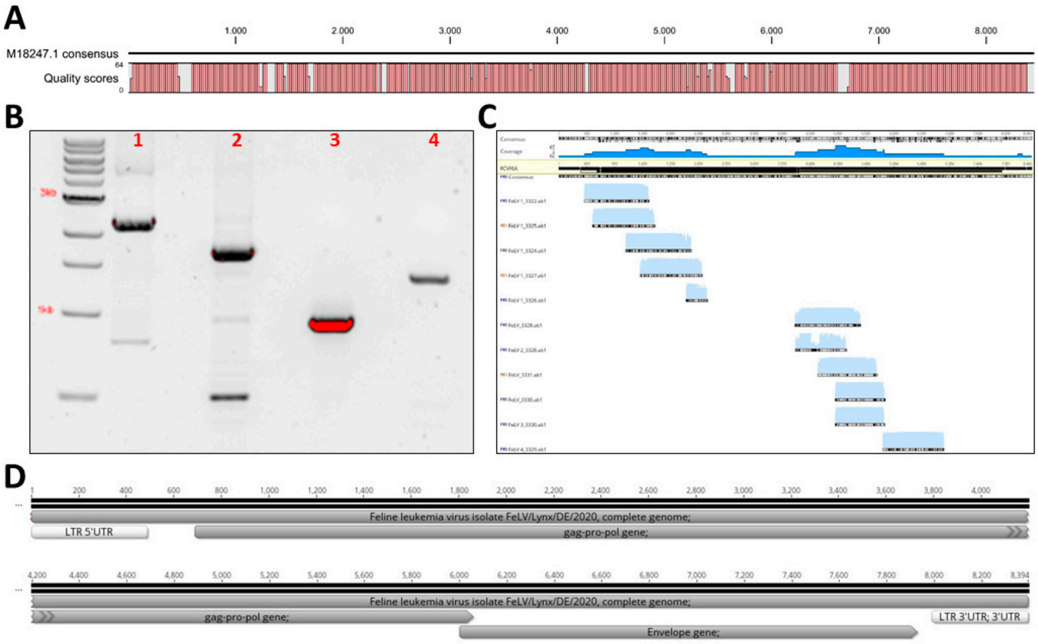

Figure S2. Detection of gap filling of FeLV genome. a) Coverage of the FeLV genome obtained following analysis of NGS data. b) PCR amplicons obtained for purposes of gap filling. c) Schematic diagram showing assembly of Sanger Sequencing FeLV PCR amplicons. d) Schematic diagram illustrating the complete recovered FeLV genome.

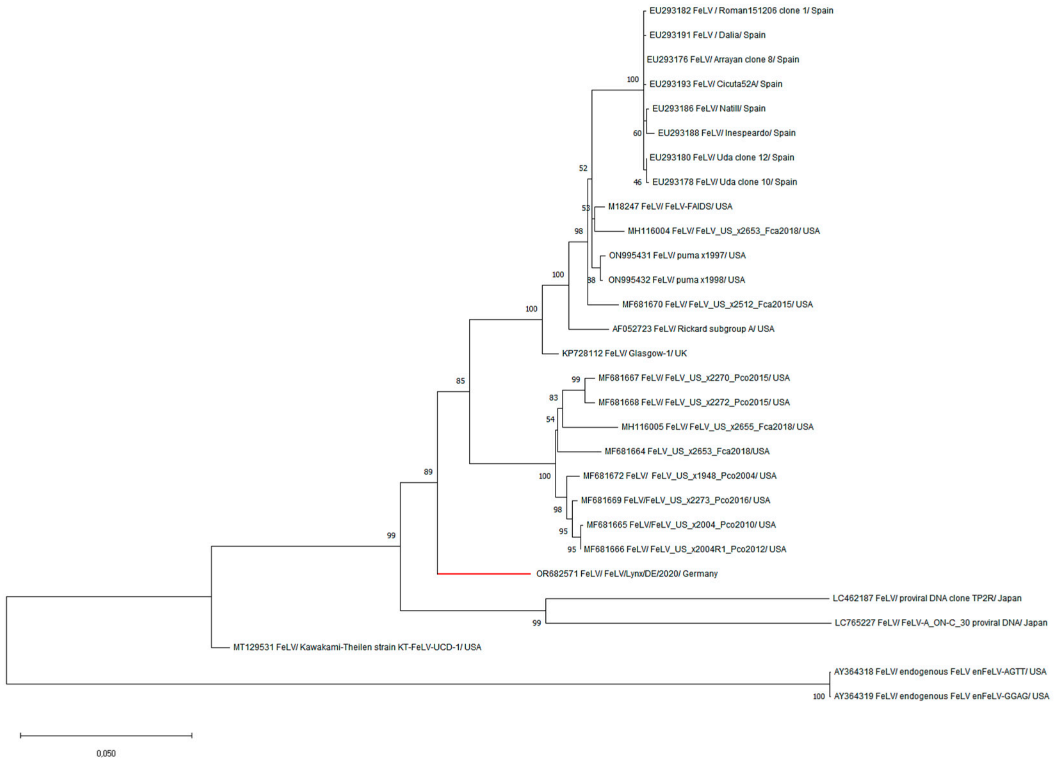

Figure S3. Phylogenetic analyses of FeLV partial sequence of envelope genes. A maximum likelihood phylogenetic tree was generated for FeLV envelope genes using HKY+G as the best fit model and 1000 bootstrap values. Bootstrap values are presented at nodes. Scale bar indicates number of nucleotide changes per site. The Gen Bank accession number, virus name, and strain indicate taxon names at the tip labels.

References

1. Allander, T.; Emerson, S. U.; Engle, R. E.; Purcell, R. H.; Bukh, J. A virus discovery method incorporating DNase treatment and its application to the identification of two bovine parvovirus species. *Proc Natl Acad Sci U S A* **2001**, *98***,** 11609-11614. https://doi.org/10.1073/pnas.211424698
2. Kalantar, K.L.; Carvalho, T.; de Bourcy, C. F. A.; Dimitrov, B.; Dingle, G.; Egger, R.; et al. IDseq-An open source cloud-based pipeline and analysis service for metagenomic pathogen detection and monitoring. *GigaScience* **2020**, *9*. https://doi.org/10.1093/gigascience/giaa111
3. Tandon, R.; Cattori, V.; Gomes-Keller, M. A.; Meli, M. L.; Golder, M. C.; Lutz, H.; Hofmann-Lehmann, R. Quantitation of feline leukaemia virus viral and proviral loads by TaqMan real-time polymerase chain reaction. *J Virol Methods* **2005**, *130*, 124-132. https://doi.org/10.1016/j.jviromet.2005.06.017
4. Katoh, K.; Rozewicki, J.; Yamada, K.D. MAFFT online service: Multiple sequence alignment, interactive sequence choice and visualization. *Brief Bioinform* **2019**, *20*, 1160-1166. https://doi.org/10.1093/bib/bbx108
5. Tamura, K.; Stecher, G.; Kumar, S. MEGA11: Molecular evolutionary genetics analysis version 11. *Mol Biol Evol* **2021**, *38*, 3022-3027. https://doi.org/10.1093/molbev/msab120
6. Erbeck, K.; Gagne, R. B.; Kraberger, S.; Chiu, E. S.; Roelke-Parker, M.; VandeWoude, S. Feline leukemia virus (FeLV) endogenous and exogenous recombination events result in multiple FeLV-B subtypes during natural infection. *J Virol* **2021**, *95*, e0035321. https://doi.org/10.1128/jvi.00353-21
7. Martin, D.P.; Murrell, B.; Golden, M.; Khoosal, A.; Muhire, B. RDP4: Detection and analysis of recombination patterns in virus genomes. *Virus Evol* **2015**, *1*, vev003. https://doi.org/10.1093/ve/vev003
8. Goettling, J.; Goeritz, F.; Jewgenow, K.; Painer, J. Serum chemistry and haematology for female Eurasian lynx (*Lynx lynx*). *Eur J Wildl Res* **2016**, *62*, 365-367. https://doi.org/10.1007/s10344-016-0990-4
9. Moen, R.; Rasmussen, J.M.; Burdett, C.L.; Pelican, K.M. Hematology, serum chemistry, and body mass of free-ranging and captive Canada lynx in Minnesota. *J Wildl Dis* **2010**, *46*, 13-22. https://doi.org/10.7589/0090-3558-46.1.13
